# Semi-guided learning tool as framework for STEM students learning: A case study for final year projects

**DOI:** 10.1007/s10639-022-11231-0

**Published:** 2022-08-01

**Authors:** María Luz Morales-Botello, Carlos Moreno Martínez

**Affiliations:** School of Architecture, Engineering and Design, Universidad Europea de Madrid, 28670 Villaviciosa de Odón, Madrid Spain

**Keywords:** Higher education, STEM, Final year project, Skills, Student perceptions, Self-efficacy

## Abstract

Communication and planning are skills expected among graduates. However, a skills deficit continues to be present among students. To address this problem, here we considered the final year project (FYP), a widely used way of working on skills in STEM degrees. Furthermore, we took into account recent research that highlights the importance of students' thoughts, beliefs, and emotions, in addition to characteristics such as self-efficacy or self-management within the learning process. Here a framework was designed around a semi-guided learning approach, with the aim of improving STEM students' skills and providing them with a work context that facilitates favorable behaviors and feelings associated with writing their FYP report. This framework, implemented in a Spanish university in Madrid, was designed to accomplish their work during restricted face-to-face work due to COVID-19. Through mixed-focus surveys, the perceptions of 55 students (male/female ratio = 4) were collected in relation to what the framework had provided them. The results showed that most of the students perceived improvement both in planning and written communication skills. The qualitative analysis also allowed us to determine the most common difficulties found among students, as well as benefits provided by the framework, among which they highlighted, greater efficiency in the writing process and help to ensure the quality of the FYP report. Our findings allow promoting beneficial results for STEM student and support the idea of considering the cognitive-emotional context of the student as a part of the environment in which to develop facilitating tools for learning and skills development.

**Supplementary Information:**

The online version contains supplementary material available at 10.1007/s10639-022-11231-0.

## Introduction

Science, Technology, Engineering and Mathematics (STEM) are growing fields which offer significant job opportunities. These industries demand technically competent graduates with strong “soft” skills, such as critical thinking, problem solving, teamwork, and written and verbal communication (Russell et al., 2005; Mardis et al., 2018; Succi & Canovi, 2020). In general, any graduate, and in particular an engineer, must be able to communicate effectively, convey their message clearly, and do so unequivocally (Nightingale, 1988; Wheeler & McDonald, 2000). In this sense, written communication is considered one of the most critical competencies for academic and career success (Sparks et al., 2014). This is evidenced in numerous studies, including surveys of stakeholders from higher education and the workforce, and in different professional areas such as accounting/finance, information technology, education, engineering, law, health, and science, among others (Moore & Morton, 2015; Riley & Simons, 2016). Despite the importance of this skill in university graduates, a deficit in it has been observed over the years (Moore & Morton, 2015). In particular, in written communication, a specific deficit has been found in spelling, punctuation marks, structuring of sentences and paragraphs, conciseness, clarity, as well as the structuring of information in documents, among others (Moore & Morton, 2015). This observed deficit is not exclusive to communication skills but is also present in other soft skills (Hurrell, 2016; Succi & Canovi, 2020).

Educational programs have the possibility of providing graduates with not only technical skills, but also enhancing the demanded soft skills (Bringula, 2015; Russell et al., 2005). There is a need to align educational programs and policies to the market demand, where the need for recent STEAM graduates with professional skills, such as effective communication, is increasing (Bringula, 2015; Mardis et al., 2018). In this context, the role of final year project (FYP) in students’ learning process and outcomes have been extensively discussed in academia and business. FYP are examples of activities in which students are producers of knowledge and not just consumers (Healey et al., 2013). Traditionally, they have been viewed as an effective means for research training, empowering learners, motivating students and promoting skills and employability (Beer et al., 2011; Bringula, 2015; Marshall, 2021; Payne et al., 2008; Russell et al., 2005).

However, facing the FYP is a challenge for undergraduate students. Students not only have to choose their project topic, which may depend on the institution’s learning program, the country’s higher education policy (Hassani et al., 2018), but also on the student’s personal preference or experience (Berndtsson et al., 2002). Students also face the challenge to decide the focus of the FYP, which can vary from a product development project, where the student applies predefined methods to a generally well-defined problem–solution situation, or a research-oriented project where the emphasis is on new knowledge creation applying research methods.

In addition, whatever the topic or focus of the FYP, the student must also face writing the FYP report. This process presents great difficulties in general and can be even more relevant in engineering students (Castelló et al., 2012). Moreover, students not only should be able to define the steps and time to convert a storyboard into an outline and afterwards into a final document, applying a formal writing style (Joshi, 2007). They should also be able to incorporate specific tasks in their plan, aligned with the principle of academic writing, such as how to establish a base hypothesis and how to prove it, steps to diagnose what they read, ways of incorporating external resources in the research tasks, etc. (Turabian, 2007; Zuber-Skerritt & Knight, 1986). Additionally, students should also be able to analyze, propose and justify alternative ways of shaping the project plan, for instance applying iterative models (Mahnic, 2012).

The writing process allows the development of these competencies in students; however, this process goes further. “*Writing process correspond to certain powerful learning strategies, since writing's permanence allows for re-examination of ideas, learners who do not write may lose many opportunities for learning*” (Emig, 1977). Some important elements stand out in this process, such as the supervision and feedback of the student’s work, and the context of the students themselves. In the development of FYP, educators can provide support in the form of supervision and control, but in order to maximize student satisfaction, the correct balance should be applied between student control versus guidance provided by the supervisor (de Kleijn et al., 2012; Moore et al., 2018). Studies of student perceptions show that the feedback on the development of the work is correlated with the effectiveness of such feedback (Lizzio & Wilson, 2008). Feedback, resilience and organization, and support network are also needs highlighted by research students or recent graduates in relation to growth in academic writing (Can & Walker, 2014; Odena & Burgess, 2015).

According to all the above, there is no doubt about the challenge, and at the same time, the learning opportunity that the FYP represents for students. But to achieve successful learning and the attainment of the required skills, the evidence indicates that not only the technical way should be considered to increase it, but also, attention should be paid to the aspects related to the student's own perceptions. In line with this, researchers working in educational settings are increasingly paying attention to what role students’ thoughts and beliefs play in the learning process (Van Dinther et al., 2011), and in particular, increasing attention has been paid to the construct "self-efficacy", understood as, “*person’s judgment of their confidence to learn, perform academic tasks or succeed in academic endeavors” (*Bandura, 1986*).* Research has shown that self-efficacy beliefs are important mediators of all types of achievement-related behaviors, such as effort and task persistence or self-regulatory strategies, among others (Meece et al., 2006). In this sense, educational programs have the possibility to enhance students’ self-efficacy (Cuthbert, 1995; Heikkilä & Lonka, 2006; Van Dinther et al., 2011; Onah et al., 2020; López-Crespo et al., 2021; Rodríguez-Jiménez et al., 2021). Furthermore, findings from recent studies also shed new light on the under-valued and frequently occluded role of positive emotions in the writing process (Sword et al., 2020).

Recent events, like the COVID-19 pandemic, have added an important overall impact on the academic community, and in particular to FYP, limiting the active involvement of educators and imposing the use of online platforms (Marinoni et al., 2020). Without forgetting the importance of the emotional impact that it caused on the students themselves and, therefore, on their performance and learning during the completion of their FYP, which was in itself a highly demanding activity.

Considering all that has been described, the context of the FYP seems to be a powerful way to increase in graduates those skills highly demanded by the industry and in which a significant deficit is still observed (Hurrell, 2016; Succi & Canovi, 2020). In addition, considering the challenge that facing the FYP represents for the students, a possible impact on their thoughts, beliefs or emotions should be taken into account. With the aim of improving STEM students' skills and providing them with a work context that facilitates favorable behaviors and feelings associated with writing their FYP report, we designed a framework around a semi-guided learning method, which was implemented for engineering students at a Spanish university in Madrid that applies project-based learning as a central element of teaching.

The purpose of this study was to investigate, through surveys, the perception of students regarding the method used around the following main aspects: (i) improvement in specific written communication and planning skills, (ii) efficiency in the process of writing their FYP report, (iii) quality of the outcome and work delivered, and (iv) if it helped them to “think outside the box”, for instance, motivating them to proactively broaden the scope of their research. This study is important, firstly, because it directly addresses improvement in written communication, a skill of recognized importance but in which students and graduates are showing deficits over the years. Secondly, this study analyzes the relationship between the provided method and the improvement in how students approach their work, if it motivates students to explore aspects such as the added value of their FYP to society and business, and if it motivates students to innovate or research more than initially planned. Thirdly, this study considers a diverse range of degrees within engineering as well as a diversity of approaches to FYP. Fourthly, this study makes it possible to identify errors and difficulties encountered by engineering students, as well as the benefits of the method provided in an emotionally complicated context in which a guide or support framework for the student can acquire greater relevance. In this way, this study provides knowledge to work more efficiently in the delicate balance between the promotion of technical skills and personal and communication skills in the context of higher education.

## Methods

### Study design

This section describes the context of the subject "Final Year Project", as well as the learning method implemented in the institution where the study was framed.

FYP has a study baseline between 12 and 18 ECTS (European Credit Transfer System). Students are expected to work independently most of the time. The student’s work is developed in collaboration with one or more tutors, through periodic review meetings. Considering the restrictions due to Covid-19, all meetings were held online. Regarding the FYP focus, some students chose product development-oriented projects, others chose research-oriented projects, and blended projects were also chosen. Some examples of the topic and focus of FYP are: “*IoT Data Analytics for the prediction of Burnout Syndrome*” (research-oriented), “*Prototype of a system for rehabilitation of the hemiplegic hand*” (product development-oriented), “*Bike user experience metering*” (blended project).

The method offered a framework that supports student learning in their academic and professional growth, as well as their personal perception of higher performance. It was aligned with the phases of the FYP creation process, enabled student self-management, and encouraged a quality-driven approach to produce a higher value result in both content and style.

**Fig. 1:**
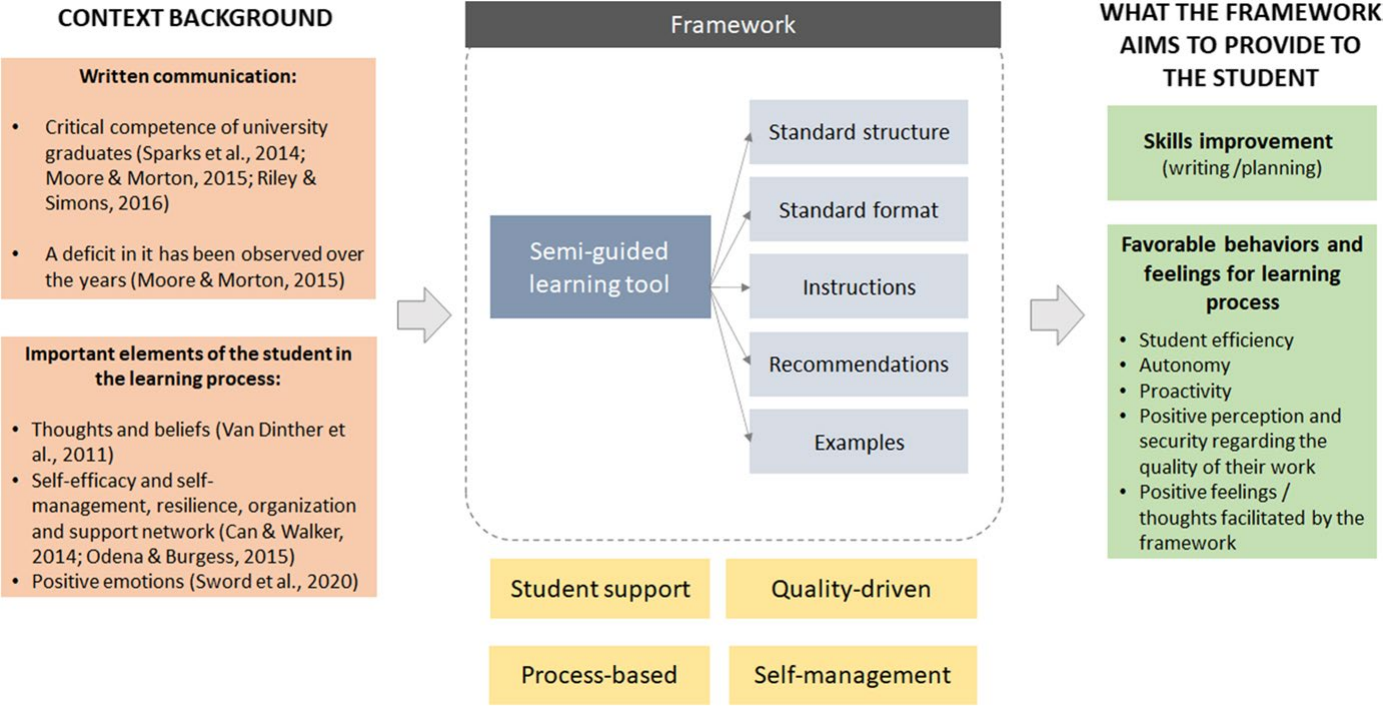
Framework context background, elements and objectives

The framework consisted of several interrelated elements. First, it offered a formal structure for the FYP report, as a working guide, proposing general and optional chapters. This structure starts with an executive summary, following with the chapters commonly used in this type of student work (Safieddine, 2016). However, students were free to structure and choose the level of detail of the main chapter. This required that the students proactively decide the depth of their FYP report content, and therefore stimulated them to plan in advance. The framework also included embedded instructions that address practical aspects of format and writing style (e.g., the appropriate use of academic references), and aimed to minimize supervision in writing, promoting student autonomy (self-management). The third element was embedded recommendations, which helped students to understand each specific section and its relationship with the work as a whole. These recommendations also encouraged student to reflect about the value of their work for companies and society in general, through sustainability and the benefit generated by the knowledge created in their FYP. Some examples of concepts underlying these recommendations were studying the feasibility of the project, defining the benefit of collaborating with companies in carrying out the project, and analyzing the degree of academic, technological or industry maturity in the field of application of their FYP. Finally, the framework proposed illustrative examples, which were intended to motivate students to enrich their FYP report by adding their own relevant examples during the creation process. Illustrative examples also provided an opportunity for the student to compare their writing, to assess clarity and conciseness. For instance, the description of general and specific objectives for all types of projects (research-oriented, product development-oriented, blended project), were extracted from relevant former FYPs and provided as illustrative examples. The context background, elements and the objectives pursued by the framework are shown in Fig. 1.

These elements were structured in a single item that was delivered to the student in the form of a FYP report template, created by two professors with different expertise and profiles (science-research, engineering-business). It was specifically designed to provide documentary support to the framework and offer foundation content and method to students during restricted face-to-face work. It was provided to students for mandatory use in the 2019–2020 academic year for their FYP report creation. Table 1 contains a summary of the template structure.

**Table 1:** Description of the template structure of the Final Year Project report

Sections	Subsections	Comments
PROJECT FRONT PAGE	-	This is the institution’s standard front-page format for FYP
SUMMARY/ABSTRACT	-	Students were asked to summarize their work in a 150 to 200-word abstract, both in Spanish and English, including keywords
Chapter 1. SUMMARY OF THE PROJECT	1.1 Context and justification 1.2 Statement of the problem 1.3 Project objectives 1.4 Outcomes 1.5 Structure of this document	The goal is to lead students to think in a general perspective, highlighting the fundamental aspects of their work. This sections also serves as a foundation in learning to write executive summaries commonly used in the business context
Chapter 2. BACKGROUND / STATE OF THE ART	2.1 State of the art 2.2 Context and justification 2.3 Statement of the problem	In this section the student can present previous experiences and knowledge, identify unanswered questions, or position their work in a business context
Chapter 3. OBJECTIVES	3.1 General objectives 3.2 Specific objectives 3.3 Project benefits	This section drives students to think in clear and measurable goals. Benefit aspects were also introduced, to widen students’ awareness of the value of their work
Chapter 4. PROJECT DEVELOPMENT	4.1 Project planning 4.2 Description of the solution, methodologies and tools used 4.3 Required resources 4.4 Budget 4.5 Feasibility 4.6 Project results	This is the main chapter of the FYP report. Freedom of choice was given to students to adapt the level of detail of the subsections
Chapter 5. DISCUSSION	-	This section is aimed to motivate students to assess their own approach and method applied in their FYP
Chapter 6. CONCLUSIONS	6.1 Conclusions of the work 6.2 Personal conclusions	This section allows students to state the FYP results and findings from their own perspective, and to add a personal point of view of their experience working on the FYP
Chapter 7. FUTURE LINES OF WORK	-	This chapter drives students to think in future expansion and potential evolution of their work
Chapter 8. REFERENCES	-	Chapter to list the references used in the FYP, in standard format
Chapter 9. ANNEXES	-	Chapter to add complementary content and additional details

### Questionnaire design/ Survey design

A survey was designed and implemented to collect the perception of the students following a mixed approach (Curry et al., 2009; Wisdom et al., 2012) in which both quantitative and qualitative information was collected.

The survey was structured into seven categories directly related to the students’ context information and the study’s points of interest. Table 2 shows the organization of the survey, including the ID of the questions grouped in each category or subcategory.

**Table 2:** Survey organization: sections, categories, subcategories, question IDs and question type (* indicates open-ended question)

Section	Category and subcategory	Question ID
1. Context information	1—General information	1*, 2, 3, 4, 4bis*, 5
	2—Areas of interest and proactivity	6, 7, 8
2. Student efficiency	3—Efficiency in FYP report writing	
	3.1—Planning and time to complete FYP report	9, 10
3. Student experience with the framework	4—The framework (template) as a semi-guided learning tool	
	4.1—Student experience	11, 12
	4.2—Template completeness	13*
	5—The framework (template) as a motivational element	
	5.1—What value does the template provide to students	14*
	5.2—Improvement in communication skills	15*, 15bis*
	5.3—Improvement in planning skills	16, 16bis*
	5.4—“Thinking bigger/outside the box”	17, 17bis*
	6—Relationship with sustainable development	18*
	7—Additional relevant information	19*

The survey included a set of close-ended questions that allowed the collection of quantitative information and a set of open-ended questions that allowed the collection of qualitative information, with a total of 23 questions. The close-ended questions were either (i) “multiple choice”, where students could select only one of the answer options, and (ii) “multiple answer”, where students could select more than one of the answer options. The open-ended questions allowed students to express themselves freely, providing a deeper level of information and capturing qualitative information in addition to quantitative information. A detailed description of the survey is included in the appendix.

Section 1 of the survey aimed to collect general information from the students related to their studies, employment situation, areas of interest, as well as their proactivity for the development of a FYP in collaboration with companies. Section 2 refers to "student efficiency" in terms of the time needed to write the FYP report. Here, we intended, on the one hand, to obtain an estimate of the time required by students to write their FYP report, from their own perception; and on the other, to quantify the students' ability to estimate that time correctly. Finally, Sect. 3 (student experience with the framework) aimed to collect the students' perception in relation to the writing phase of their FYP report and the effect of the framework on their skills. At the end of this section, the survey included a question that allowed us to explore the student's perception of the concept of sustainability, specifically in the context of their FYP.

### Sample

The participants (n = 55) were students who presented their final degree project in the 2019–2020 academic year, in the degrees of IT Engineering (ITE), Biomedical Engineering (BE), Telecommunications Systems Engineering (TSE) and double degrees in Engineering & Business Administration (IT&BA). Students used three possible learning formats (online, onsite, hybrid). Students were assigned a numerical identification code to guarantee anonymity.

### Data collection

The study objective and survey details were sent via email to the head of department, academic coordinator and coordinators of the degrees involved in the study to get formal approval. The coordinator of final degree projects informed the participants and sent a link to the survey through announcements sent from the subject's virtual campus. The survey questions were developed and published using Google Forms (https://docs.google.com).

### Data analysis

The data analysis consisted of a quantitative analysis of the responses to the close-ended questions and a qualitative analysis of the responses to the open-ended questions. The results of the quantitative analysis are presented as number and percentage of respondents who choose a certain answer option. In addition, Kruskal–Wallis test was used to determine if there were differences in the improvement in skills (questions 15 and 16) among students of different degrees. Affirmative answers to these questions were coded as 1 and negative answers were coded as 0, for the application of the non-parametric test. Six tests were carried out to make comparisons between degrees for each skill analyzed. Differences were considered significant at *p* < 0.05 and all tests were performed with Statistics and Machine Learning Toolbox in Matlab (version 2020b, the Mathworks).

Regarding the qualitative analysis, a content analysis of the answers was performed. This analysis showed the existence of common elements, which were categorized according to key themes. This analysis included the extraction of key themes from students' perceptions about what was difficult or easy for them to complete in the template, and changes they suggested (question 13), and the value provided by the template (question 14). The joint analysis of the answers to questions 11, 12, 13 and 14 it was used as a way of exploring and investigating the difficulties that the students encountered when writing the different sections, as well as the benefits found with the method, in order to use this feedback from the students to improve the method in future revisions. Qualitative analysis was also performed in relation to specific improvements in communication skills (question 15bis) and planning skills (question 16bis). As well as perceptions about how the template has helped them to delve into aspects not previously considered (question 17) and relevant information not mentioned in previous questions (question 19). The answers to questions 14 and 19 allowed us to extract the general perception of the students regarding the use of the template, which was categorized as positive, negative, neutral or both (positive/negative). In addition, from the answers to question 19 we were also able to extract and categorize the changes proposed by the students and how to apply them.

The results of these analyses are shown in the next section following the structure described in Table 2.

## Results

### Section 1: Context information

#### Category 1: General information

A total of 55 students of various engineering degrees, ages, and genders completed the survey. The average age was 26 years. The male/female proportion was 80%/20%. Nearly half of the students declared that they currently have a full-time job. Further details of the students’ context information are provided in Table 3.

**Table 3:** Responses of the students to general context information

Question	Response
01—Age	21–46 years (average 26.6 ± 6.06)
02—Gender	Female (n = 11, 20%) Male (n = 44, 80%)
03—Are you currently working or doing internships in companies?	Full-time job (n = 27, 49%) Practicum (n = 7, 13%)
04 and 04bis—Degree you are studying	IT Engineering (ITE) (n = 33, 60%) Biomedical Engineering (BE) (n = 7, 13%) Telecommunications Systems Engineering (TSE) (n = 8, 15%) Double degree (IT Engineering & Business administration, ITE&BA) (n = 7, 13%)
05—Modality in which you study the degree	On campus (n = 32, 58%) Online (n = 19, 35%) Hybrid (n = 4, 7%)

#### Category 2: Areas of interest and FYP collaboration with external companies

**Fig. 2:**
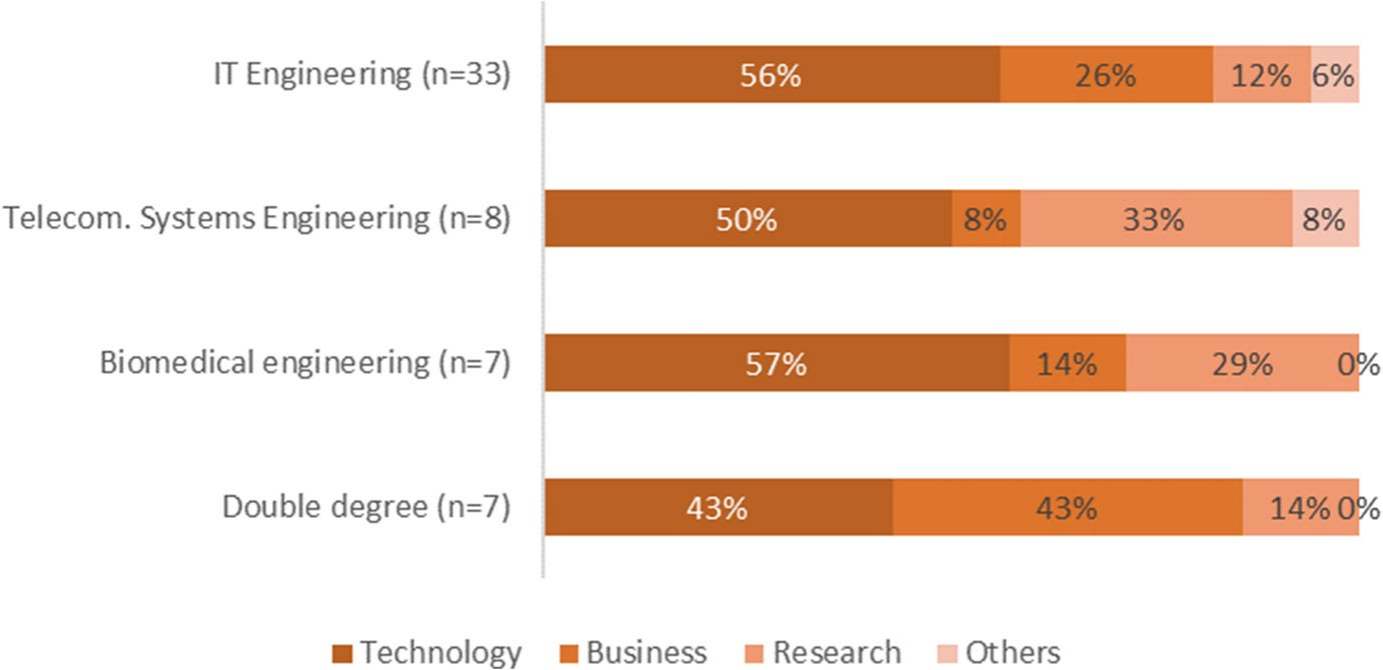
Areas of interest (n = number of students per degree, % responses for each area per degree)

Related to students’ greatest areas of interest (multiple-answer question 6), technology was selected 44 times (80% of students), followed by business-related topics (n = 21, 38%), research related work (n = 14, 25%), and others (n = 4, 7%). The reasons that the student gave for their interests (multiple-answer question 7), related to “their degree” was selected 42 times (76% of students), related to “their professional experience” (n = 26, 47%), and “others” (n = 2, 4%). The relationship of these areas of interest and the degrees that the students were studying is shown in Fig. 2.

Related to the proactivity in proposing a collaboration with an external company (question 8), most student expressed that it was not in their plans and did not propose any collaboration (84%, n = 46).

### Section 2: Student efficiency

#### Category 3: Efficiency in FYP report writing

Question 9 captured the actual time students spent writing their FYP report and question 10 captured the initially planned time, based on three response options (less than 10 h, between 10 and 30 h and more than 30 h). Related to FYP planning estimation, the results showed that 40 out of 55 students (73%) matched their initially planned effort. All the remaining students (n = 15) dedicated more time to complete the report than initially expected. Related to the actual time to complete the FYP writing, no student spent less than 10 h; 20% (n = 11) of the students reported having spent between 10 and 30 h; and the majority (80%, n = 44) reported having dedicated more than 30 h.

### Section 3: Student experience

#### Category 4: The framework (template) as a semi-guided learning tool

In questions 11 and 12, students were asked which chapters were the most difficult to write, and which was the easiest to complete, respectively. "Background / State of the art" and "Project development" were the most complex chapters. Whereas “Summary”, “Objectives”, “Conclusions” and “Future lines of work” were perceived as simpler chapters to write, while the percentage of students who described them as complicated was considerably lower. “References” and “Annexes” were also described as simpler by most of the students who mention these chapters. Table 4 summarize these results.

**Table 4:** Chapters perceived by students as the most difficult or the easiest to write (n = number of responses mentioning each chapter)

Chapter	Difficult (n)	Easy (n)
Summary	9	**24**
Background / State of the art	**26**	12
Objectives	12	**23**
Project development	**25**	**23**
Discussion	**13**	**10**
Conclusions	6	**22**
Future lines of work	3	**24**
References	5	**15**
Annexes	1	**7**
None	1	0

In question 13 (open-ended), the students were asked to clarify the answer to questions 11 and 12, and if they would change something of the template. The content analysis of the responses showed that the topics most highlighted by students were the difficulty to differentiate between context, justification, problem statement and state of the art and understanding how and why to write an executive summary. In addition, 20 out of 55 students indicated that they would not change anything in the template, and that it was easy to understand and use. Examples of student responses and their corresponding key topics are shown in Table 5.

**Table 5:** Key topics mentioned by students about difficulties encountered, and proposed changes to the template (n = number of responses related to each topic)

Key topics	n	Representative response
Difficulty to differentiate between context, justification, problem statement and state of the art	11	“Sometimes ideas from one part of my report overlapped in another and it was difficult for me not to repeat the same ideas”
Difficulty to understand how and why to write an executive summary	9	“The template was very good but due to its structure there were concepts that were repetitive. I would change the initial summary for a general introduction to the project”
Preference for a predefined structure for the main chapter (chapter 4)	5	“It was a bit difficult to complete the development because it was the only section whose structure was not very defined, and I had to define it”
Preference of free format vs. predefined template	5	“I would propose a flexible number of chapters”
Clarification of the project goal structure	2	“… at some points it seemed to me that it repeated a concept, such as project objectives, general and specific objectives. I did not really know very well where to cover it and I had the feeling that I was repeating the same thing with different words. Everything else seemed good to me”
Project viability	1	“I don’t understand clearly what project viability means”
Discussion section suitability in non-research projects	1	“The discussion section should not apply for product development work; it should only apply for research"
Economical aspects of the project (e.g., project budget)	1	“I would make the budget optional since there are research projects where it may have less relevance”

#### Category 5: The framework (template) as motivational element

Students were asked in question 14 to describe the value provided to them by the template. Our analysis of the responses to this question revealed that important aspects such as structure, guidance, efficiency, and quality were frequently highlighted by students. Table 6 shows complete information of all the key aspects found.

**Table 6:** Key aspects identified by students about the value provided by the template (n = number of responses related to each topic)

Key aspects	n	Representative response
Helps to structure the thesis	34	“It has helped me to structure the content”
A guide for the work	33	“It has helped me to structure the content of the thesis, gaining coherence and order. In addition, the explanation of each of the sections has been very useful to me”
More efficiency	15	“Speed ​​up writing, and avoid moments when one can go blank”
Quality assurance	13	“It helps me to ensure the quality of the memory”
Global view of the project	8	“Global vision of everything that the project encompasses”
Think out of the box	7	“It makes me think of aspects that I had not planned, although I have also added some that did not appear in the template”

**Fig. 3:**
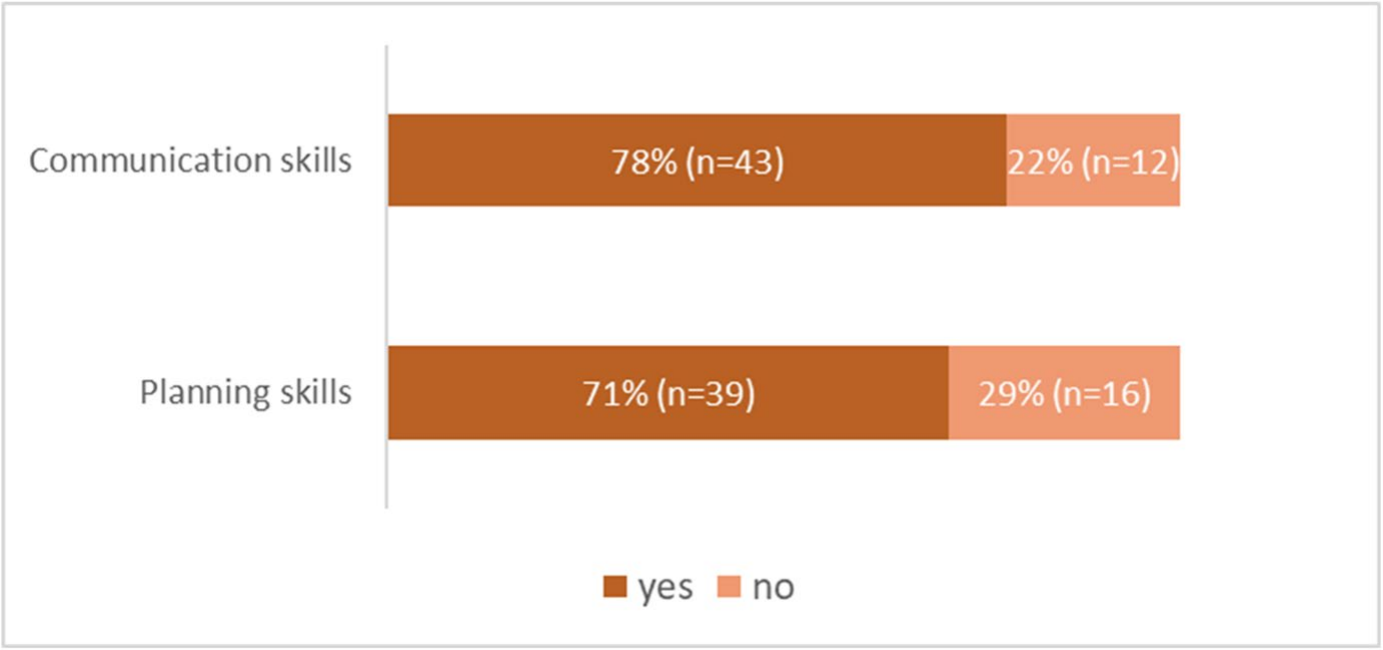
Students’ global perception of their improvement in communication and planning skills (total responses n = 55)

Regarding the perception of whether the use of the template improved written communication and planning skills (questions 15 and 16), most of the students stated that they had improved in both competencies (Fig. 3). No significant difference was found in the improvement of written communication skills among students of the four different degrees (Kruskal–Wallis test: ITE(n = 33) vs BE(n = 7): *p* = 0.9743; ITE(n = 33) vs TSE(n = 8): *p* = 0.9517; ITE(n = 33) vs ITE&BA(n = 7): *p* = 0.9743; BE(n = 7) vs TSE(n = 8): *p* = 0.8788; BE(n = 7) vs ITE&BA(n = 7): *p* = 1; TSE(n = 8) vs ITE&BA(n = 7): *p* = 0.8788). Regarding the perceived improvement of planning skills, no significant differences were found in the comparison between most of the degrees, except in the comparison between IT engineering and double degree (Kruskal–Wallis test: ITE(n = 33) vs BE(n = 7): *p* = 0.9836; ITE(n = 33) vs TSE(n = 8): *p* = 0.8039; ITE(n = 33) vs ITE&BA(n = 7): *p* = 0.0421; BE(n = 7) vs TSE(n = 8): *p* = 0.7617; BE(n = 7) vs ITE&BA(n = 7): *p* = 0.0908; TSE(n = 8) vs ITE&BA(n = 7): *p* = 0.4804).

**Fig. 4:**
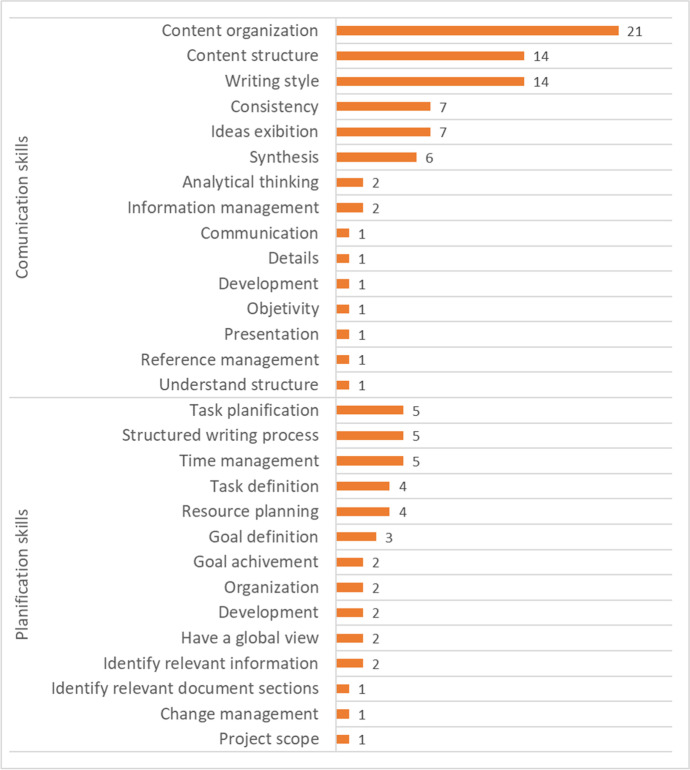
Perceived improvement in specific communication and planning skills (n = number of responses mentioning each topic)

The main aspects of improvement highlighted by students among written communication skills (question 15bis) included organization and content structure, as well as writing style, consistency, presentation of ideas and synthesis capacity. Related to planning skills, the most mentioned topics were task planning, structured writing process and time management, followed by task definition and resource planning. The full list of topics mentioned by the students related to improving communication and planning skills are listed in Fig. 4.

**Fig. 5:**
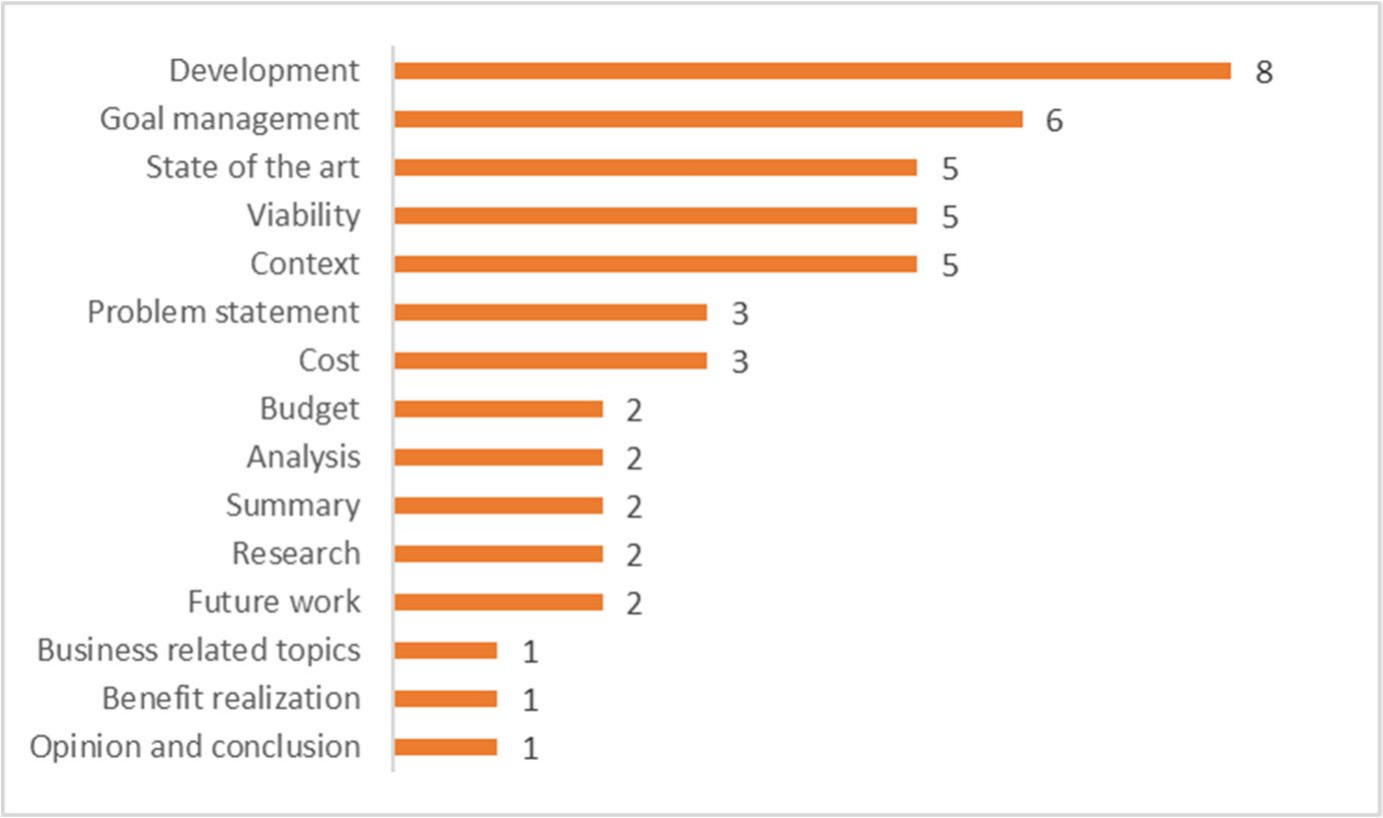
Perceived improvement in proactivity due to the template (n = number of students mentioning each aspect)

In addition, more than 70% (n = 39) of the students stated that the template motivated and helped them to investigate deeper in specific topics which were not initially planned (question 17). The most mentioned aspects were to include a chapter dedicated to discussion and conclusions on the work done, to produce a more detailed description of the future work, to consider project economics and viability, to enhance the objective definition by adding specific goals and subgoals, and also to write a more comprehensive chapter about the state of the art (question 17bis). Figure 5 contains the details on what specific aspects were mentioned by the students.

#### Category 6: FYP and sustainable development

The general opinion of the students about their project contribution to sustainable development was captured in question 18. About half of the students felt that their FYP did not contribute to this aspect (n = 31, 56.4%).

#### Category 7: Additional relevant information provided by students

From the joint analysis of the responses to the last question of the survey (question 19) and question 14, it was possible to extract a general perception of the students about the use of the template. We found that the responses of 90% (n = 49) of the students could be categorized as positive (e.g., “*In general, this template has helped me to further develop the ideas that I was already carrying out. I am very happy with the result of having applied this template to my memory”)*, 5% of them (n = 3) were categorized as both positive and negative (e.g., “It s*peeds ​​up writing, and avoids moments when one can go blank*” (positive perception) / “*Initial confusion, there seems to be some initial parts repeating*” (negative perception), and finally, 5% (n = 3) of the responses in which no type of perception (positive or negative) could be appreciated and were categorized as neutral responses (e.g., “*I have no additional comments*”). No student gave an answer that could be categorized as purely negative.

In addition, the responses to question 19 contained students' thoughts and suggestions for improvement regarding the content and how to use the template. Table 7 summarizes in detail the analysis of the students’ responses.

**Table 7:** Changes proposed by students to the template (n = number of students mentioning each aspect)

Student proposal	*Summary of students’ proposals*
Get additional guidance on how to use and apply the template (n = 6)	More support on how to use tables and figures index More reference examples More support on what type of images and tables are relevant Better explanation of why and how to write some chapters (e.g.*,* Results) Better explanation of how to write the project summary
Have more flexibility to adapt the template to their own style (n = 5)	To be able to change structure and indentation levels To have more format choices, like Latex Be able to provide supplementary digital content by any means
Have different templates depending on the FYP focus: research or product development project (n = 5)	E.g., “user manual” is only for product-focused projects Include a glossary section for technical projects Add requirements and use cases for FYP with a focus on product development
Structural changes to the template (n = 3)	Add an acronym section
More student involvement in template usage (n = 1)	Students should be able to propose changes to FYP template

## Discussion

This study presents the perception of students regarding a learning method, around the following main aspects: (i) improvement in specific written communication and planning skills, (ii) efficiency in the process of writing their FYP report, (iii) quality of the outcome and work delivered, and (iv) if it helped them to “think outside the box”, for instance, motivating them to proactively broaden the scope of their research. The method offered a framework that supports student learning in their academic and professional growth, as well as their personal perception of higher performance. The framework consisted of several interrelated elements structured in a single item that was delivered to the student as a semi-guided learning tool in the form of a template used for the writing of the FYP report.

All study participants were engineering degree students at the same higher education institution. The average age was 26 years, with more than half of them having some type of work experience, including full-time workers.

Regardless of the different degrees and student profiles, a common template for FYP report writing has been used. Since, beyond the particularities or requirements that, as future engineers, they need to use in the writing of their project proposals, they need above all basic and common communication skills.

Among the necessary skills in an engineer is the correct estimate of the time required to perform a task. However, it seems common for students to underestimate such effort (Mahnic, 2012). Here, most of the students were able to plan correctly the time required for writing the FYP report. And while we cannot establish a causal relationship between the method and this success, we must not reject a possible relationship either.

In this process of writing the FYP report, the students encountered some important difficulties which were revealed with the joint analysis of the answers to questions 11, 12 and 13. The most frequent were the wording and distinction between “context”, “justification”, “problem statement” and “state of the art”, as well as the wording of the discussion and development project; followed by the difficulty to perform content synthesis at different levels. These results are not overly surprising because properly organizing and synthesizing the content in these sections requires cognitive effort and higher-level writing skills (Kellogg, 2008). However, considering that frequent difficulty, it would probably be positive for the students, a future modification of the template for to reinforce the guide (instructions and recommendations) in all the chapters, but especially in those highlighted by the students as the most difficult (Tables 4 and 5). Although these difficulties are not exclusive to students of scientific areas (Maznun et al., 2017; Rahmat et al., 2017), for these students the composition strategy, seen as a “thinking” process where the writer dedicates as much attention to the content as well as to composing it, is less prevalent than in non-scientific writers (Brown, 2001). Here the role of the instructors is very relevant since this difficulty entails a greater margin for learning and work of this skill, which can be facilitated with adequate support. However, this instructor support should not be understood as an excess of guidance, since it is well known that many learning styles emphasize less instructor guidance and more responsibility of the student in the learning process (Moore et al., 2018; Roberts et al., 2018).

The use of the framework for the writing of the FYP report provided students with a series of benefits, which are described and discussed below. The support to structure the document and the guide-explanations throughout it, were the values ​​most frequently highlighted by the students. In addition, a considerable number of students highlighted that the template contributed to a greater efficiency in the writing process and helped them to ensure its quality. Finally, “global view of the project” and “think out of the box” were also mentioned as benefits contributed by the template. Furthermore, given the frequent difficulty that students encounter in the writing process (Rachal et al., 2007), it can be considered that a good result has been obtained here in terms of the improvement that most students have perceived in their skills. The main aspects of improvement highlighted by students among written communication skills included organization and content structure, as well as writing style, consistency, presentation of ideas and synthesis. These results are important because written communication is considered one of the most critical competencies for academic and career success (Sparks et al., 2014). Despite this, a deficit in these skills has been observed over the years (T. Moore & Morton, 2015). This deficit reflects, to some extent, a failure in the learning process. Although the student has direct responsibility in this process, so does the academy. From this perspective, we must ask ourselves how we can contribute to reversing this deficit. In this sense, and in the context of engineering students, there may be an imbalance between the enhancement of engineering technical skills and communication skills. It is possible that we are experiencing the consequences of a mismatch between what we give and what we require the students (Jackson et al., 2006). Regarding planning skills, the majority of students stated that they improved planning skills due to the template and that it helps as a guide for the work. However, students also mention that the most difficult chapters to write are related to core research and project planning. In this sense, it is necessary to rethink the process of writing the FYP report from the learners’ perspective, to search for additional learning methods which could improve students’ abilities to manage the development of conceptual work merged with empirical and task-oriented activities (Zuber-Skerritt & Perry, 2002).

Generally, the FYP is seen as a way to promote skills and motivate students towards learning (Marshall, 2021). Taking advantage of this context, promoting actions aimed at improving students' skills, can lead to a synergistic outcome with greater overall benefit for the student. In this sense, it is crucial to also consider the cognitive-emotional context of the student within their learning process. Thoughts, beliefs, positive emotions, self-efficacy, and self-management, among other aspects, have an impact on the success of student learning (Cuthbert, 1995; Heikkilä & Lonka, 2006; Van Dinther et al., 2011; Singer et al., 2020). In addition, according to many learning styles, an important weight has been placed on the autonomy of the student, but it seems important for successful learning that they develop that autonomy within the framework of an adequate guidance. The learning method used in this work seems to have offered the student that framework that allows them to develop or enhance their skills with certain guarantees and go further within the learning process. Having a predefined structure for the FYP report, as well as instructions for writing each section may have positively contributed to their emotions and perceptions about their writing performance. In this sense, it has been seen that writing strategies based on outlining are positively related to enjoyment whereas students who developed content and structure while writing have a more negative experience of the writing process (Torrance, 2000). Students also commented on the positive effect the template had on their motivation to work harder than initially expected (both on depth of detail and on additional content), and to think in alternatives ways of working the FYP. But students may not connect this perception with innovation and innovation capabilities. No student mentioned “innovation” in any comment in the survey. This suggests that the learning process in a FYP and the report writing should emphasize how engineering students apply and manage any of the different innovative processes to increase the value of their work as suggested by Salerno et al. (2015).

Additionally, more than half of the surveyed students stated that they did not believe that their work contributed to sustainable development. This result allowed to have a first insight into the degree of student awareness related to sustainability, and indirectly, it allowed to get an overview of the percentage of students who included the concept of sustainability in their project approach. Taking this result as preliminary, this could enable future works specifically related to sustainability and FYP.

As the last important result to mention, we found that 90% of the students expressed a positive perception about the use of the template. This result does not reflect rigorously the students’ satisfaction with the method, but it does provide a secondary perception of it, and can also be considered with the improvements perceived by the students, when evaluating the method.

Finally, although the benefits provided by the learning method were present in a high percentage of respondents, we must mention as a limitation of the study the subjective nature of the responses. This does not contradict the results; in fact, perception studies are also used as a starting point for the incorporation/evaluation of methods in education (Aparicio et al., 2018). However, considering quantitative measures of skills will provide additional insight into the results. In this sense, future studies should focus on an improvement of the method, within the teaching–learning context through the FYP: (i) incorporate the use of more rigorous competency assessment metrics, before and after using the method; (ii) include controlled test (the group with the proposed template versus the group without the proposed template); (iii) considering feedback from students, (iv) adding feedback from FYP tutors, and (v) integration into the evaluation process; (vi) investigate into greater extend the possible relationship between planning success and greater work detail, to obtain insight into content design for STEM project based learning; and (vii) implementing the framework on education technology, which could provide planification features for the work plan design, automated monitoring of the outcomes, and provide specific writing recommendations for each student for the most difficult chapters in FYP writing thru artificial intelligence capabilities.

## Conclusion

The elements that intervene in the success of learning and acquisition of skills are numerous and highly complex. Recent studies still show the existence of deficits among graduates in classical skills considered essential, such as many aspects of written communication. Likewise, characteristics such as self-efficiency, self-management, thoughts, beliefs, and emotions of the students, have gained strength as elements of great importance within the student's learning process. Framed in this context, in this work, we focused on the creation of a framework, aimed at STEM students during the writing of their FYP report, that contributes to an improvement of written communication and planning skills, providing the student with a semi-guided work environment that facilitates the enhancement of their self-management, self-efficiency and positive perceptions of their own work. The results showed that a clear improvement was perceived by most of the students in fundamental aspects of written communication and planning skills during their FYP report development. The method contributed, in a considerable number of students, to a greater efficiency in the writing process and offered them security regarding the quality of the writing. The students also highlighted the influence of the framework on their motivation to work more than initially expected and to think in alternative ways of working the FYP. Our results support the idea of ​​the adequacy of offering students a framework that serves as a guide and provides autonomy within a space of trust where they can develop and enhance their skills. This work allows us to think in the benefits for students of this type of method and reminds us of the importance of promoting and monitoring fundamental competencies of students.

## Supplementary Information

Below is the link to the electronic supplementary material.

Supplementary file1 (PDF 93 KB)

## Data Availability

The datasets used and/or analysed during the current study are available from the corresponding author on reasonable request.
