# Supplementary material for: Semi-guided learning tool as framework for STEM students learning: A case study for final year projects
Source: Educ Inf Technol (Dordr). 2022 Aug 1;28(2):1535–57. doi: 10.1007/s10639-022-11231-0 (PMC9340688; doi:10.1007/s10639-022-11231-0)
Supplement: Supplementary file 1 — Supplementary file1 (PDF 93 KB) [file 10639_2022_11231_MOESM1_ESM.pdf]

Appendix

Questionnaire details (specific questions, response type and response options)

Question id	Response type and response options
01 - Age	Open-ended
02 - Gender	Multiple choice<br>· Male/Female
03 - Are you currently working or doing internships in companies?	Multiple answer<br>· I am working in a company<br>· I am doing an internship in a company<br>· I am studying
04 - Degree you are studying	Multiple choice<br>· Degree in Computer Engineering<br>· Degree in Biomedical Engineering<br>· Degree in Telecommunication Systems Engineering<br>· Double degree
04bis - Indicate what double degree you are studying (Respond only if "Double degree" was selected in response to question 04)	Open-ended
05 - Modality in which you study the degree	Multiple choice<br>· On-line<br>· Face-to-face<br>· Semi-face-to-face
06 - What are your areas of greatest interest?	Multiple answer<br>· Technological in nature: web development, app development, IT infrastructure, Big data, artificial intelligence systems, etc.<br>· Related to business: business models, digital transformation, products and services, etc.<br>· Research in nature: analysis and studies of models and theories, study of algorithms, etc.<br>· Other (please provide additional information)

07 - Why are these areas the most interesting?	Multiple answer<br>· It is related to my degree<br>· It is related to my professional experience<br>· Other
08 - Have you proposed developing the FYP in collaboration with a company?	Multiple choice<br>· Yes / No
09 - How many hours have you spent writing your memory?	Multiple choice<br>· Less than 10 hours<br>· Between 10 and 30 hours<br>· More than 30 hours
10 - How many hours did you initially plan to spend writing your memory?	Multiple choice<br>· Less than 10 hours<br>· Between 10 and 30 hours<br>· More than 30 hours
11 - Which chapters have been the most difficult to complete?	Multiple answer<br>· Summary<br>· Background / State of the art<br>· Objectives<br>· Project development<br>· Discussion<br>· Conclusions<br>· Future lines of work<br>· References<br>· Annexes<br>· Other
12 - Which chapters have been the easiest to complete?	Multiple answer<br>· Summary<br>· Background / State of the art<br>· Objectives<br>· Project development<br>· Discussion<br>· Conclusions<br>· Future lines of work<br>· References<br>· Annexes<br>· Other

13 - Indicate what was difficult or easy for you to complete and if you would change any aspect or chapter of the template	Open-ended
14 - What has the template provided to you? Examples:” It has been useful to guide the writing of the report. It helps me to ensure the quality of my memory. I can focus on the content, and less on the format."	Open-ended
15 - Do you think that by using the template you have improved your communication skills (organizing content, writing style, etc.)?	Multiple choice<br>· Yes / No
15bis - Indicate which communication skills you consider having improved (Respond only if “Yes” was selected in response to question 15)	Open-ended
16 - Do you think that by using the template you have improved your ability to plan (set objectives, define tasks, verify compliance with objectives, etc.)?	Multiple choice<br>· Yes / No
16bis - Indicate which planning skills you consider have improved (Respond only if “Yes” was selected in response to question 16)	Open-ended
17 - Do you think that the use of the template has motivated you to delve into some aspect of your project, initially not foreseen (context, justification, relationship with business or society, feasibility, costs, discussion, and critical analysis of its results, etc.)?	Multiple choice<br>· Yes / No
17bis - Indicate what aspect has led you to delve deeper (Respond only if “Yes” was selected in response to question 17)	Open-ended

18 - Do you think that your FYP considers or contributes to sustainable development (social, economic or environmental)? Formulate it in your own words, outside of a formal definition of sustainable development.	Open-ended
19 - Indicate something that you consider relevant in relation to the memory template, which has not been mentioned in any previous question.	Open-ended
